# Towards gene therapy against HIV-1: new therapeutic target in gag RNA accessible to ribozymes and RNA interference molecules

**DOI:** 10.1186/1471-2334-14-S2-O19

**Published:** 2014-05-23

**Authors:** A Gatignol, RJ Scarborough, MV Lévesque, E Boudrias-Dalle, IC Chute, SM Daniels, RJ Ouellette, J-P Perreault

**Affiliations:** 1 Lady Davis Institute for Medical Research, McGill University, Montreal, Canada; 2 Department of Biochemistry, University of Sherbrooke, Sherbrooke, Canada; 3 Atlantic Institute for research on cancer, Moncton, Canada

## Aim

Antisense molecules targeting HIV-1 RNA have the potential to be used as part of combination gene or drug therapy to treat HIV-1 infection to reach a functional or complete cure. Only a small number of extremely active molecules currently exist and a treatment option has not yet been identified in clinical trials. We have previously developed new hepatitis delta virus (HDV)-derived ribozymes (Rzs) called "switch on-off adaptor" (SOFA) to target HIV-1 RNA [1]. Our aim is to develop highly active RNA-based molecules with complementary mechanisms, which are able to reach a large number of HIV-1 variants.

## Methods

We screened HIV-1 RNA to identify conserved target sites for new HDV-Rzs [2]. We designed new SOFA-HDV-Rzs against the Gag RNA and developed a rapid test to evaluate the inhibition of HIV-1 production. We designed small interfering (si) RNAs targeting the same region and tested their activity on HIV-1 replication.

## Results

We identified 13 conserved regions in the gag RNA and constructed the corresponding Rzs. We transfected HEK293T cells with these Rzs and HIV-1 molecular clones. We identified one Rz that was particularly efficacious. We then constructed siRNAs and short hairpin (sh) RNAs targeting the same sequence. The shRNA was very active against HIV-1 clades B, C and A/G. Neither the Rz, nor the shRNA disturbs the cellular transcriptome, suggesting no toxicity. In lymphocytic cell lines, both the Rz and the shRNA inhibit long-term HIV-1 replication [2].

## Conclusions

We identified new SOFA-HDV-Rz, siRNA and shRNA targeting HIV-1 Gag RNA. The shRNA is as active as the only shRNA that has advanced to clinical trials and targets more strains. Long-term inhibitory activity of these molecules shows that this site is particularly accessible to other antisense molecules. These molecules have a high potential to be used in combination gene therapy or as drugs with appropriate delivery methods.
